# Cortical responses to salient nociceptive and not nociceptive stimuli in vegetative and minimal conscious state

**DOI:** 10.3389/fnhum.2015.00017

**Published:** 2015-01-29

**Authors:** Marina de Tommaso, Jorge Navarro, Crocifissa Lanzillotti, Katia Ricci, Francesca Buonocunto, Paolo Livrea, Giulio E. Lancioni

**Affiliations:** ^1^Basical Medical Science, Neuroscience and Sensory System (SMBNOS) Department, Bari Aldo Moro UniversityBari, Italy; ^2^Fondazione San Raffaele-Presidio Ospedaliero di Riabilitazione ad Alta SpecialitàBrindisi, Italy

**Keywords:** disorders of consciousness, pain, event related potentials, non-nociceptive salient stimuli, nociceptive laser stimuli

## Abstract

**Aims:** Questions regarding perception of pain in non-communicating patients and the management of pain continue to raise controversy both at a clinical and ethical level. The aim of this study was to examine the cortical response to salient visual, acoustic, somatosensory electric non-nociceptive and nociceptive laser stimuli and their correlation with the clinical evaluation.

**Methods:** Five Vegetative State (VS), 4 Minimally Conscious State (MCS) patients and 11 age- and sex-matched controls were examined. Evoked responses were obtained by 64 scalp electrodes, while delivering auditory, visual, non-noxious electrical and noxious laser stimulation, which were randomly presented every 10 s. Laser, somatosensory, auditory and visual evoked responses were identified as a negative-positive (N2-P2) vertex complex in the 500 ms post-stimulus time. We used Nociception Coma Scale-Revised (NCS-R) and Coma Recovery Scale (CRS-R) for clinical evaluation of pain perception and consciousness impairment.

**Results:** The laser evoked potentials (LEPs) were recognizable in all cases. Only one MCS patient showed a reliable cortical response to all the employed stimulus modalities. One VS patient did not present cortical responses to any other stimulus modality. In the remaining participants, auditory, visual and electrical related potentials were inconstantly present. Significant N2 and P2 latency prolongation occurred in both VS and MCS patients. The presence of a reliable cortical response to auditory, visual and electric stimuli was able to correctly classify VS and MCS patients with 90% accuracy. Laser P2 and N2 amplitudes were not correlated with the CRS-R and NCS-R scores, while auditory and electric related potentials amplitude were associated with the motor response to pain and consciousness recovery.

**Discussion:** pain arousal may be a primary function also in vegetative state patients while the relevance of other stimulus modalities may indicate the degree of cognitive and motor behavior recovery. This underlines the importance of considering the potential experience of pain also in patients in vegetative state and to appropriately assess a possible treatment also in those patients.

## Introduction

Pain and pleasure are inherently subjective experiences that persons can communicate to others verbally and non-verbally (Merskey et al., 1994). In non-communicative, severely brain damaged patients, however, we can only infer those experiences by evaluating behavioral responses to external stimuli. To date, questions regarding perception and management of pain in non-communicating patients continue to raise controversy both at a clinical and ethical level (Demertzi et al., 2013). Neuroimaging studies have shown that disorders of consciousness are characterized by distinct cerebral patterns in response to sensory stimulation (Laureys et al., 2002; Kassubek et al., 2003; Boly et al., 2008; Zanatta et al., 2012). Laureys et al. (2002) directly investigated the central processing of nociceptive stimuli by using positron emission tomography (PET). In 15 VS patients, they found no evidence of noxious stimulation-related downstream activation beyond primary somatosensory cortex. More importantly, functional connectivity assessment showed that the observed cortical activation subsisted as an island, dissociated from the pain matrix and the higher-order cortices that are currently thought to be necessary for conscious awareness. Additional activation of secondary somatosensory and insula cortices in VS and coma patients, implying the possibility of affective experiences of pain, was also reported (Kassubek et al., 2003; Zanatta et al., 2012). Using nociceptive stimuli, Boly et al. (2008) showed brain activation similar to controls in five MCS patients with involvement of the thalamus, the primary somatosensory cortex, the secondary somatosensory cortex or insula, the posterior cingulate cortex/precuneus, and the anterior cingulate area; the latter area is known to be linked to pain unpleasantness perception and to cognitive and affective processing of pain.

Behavioral assessment methods also may be used to detect pain perception, to signal the need for intervention, and to evaluate treatment effectiveness as numerous pain scales have been developed for non-communicative patients (Herr et al., 2006). In this context, the Nociception Coma Scale (NCS) has been recently validated and revised to assess and detect behavioral signs of pain in patients with Disorders of Consciousness (DOC) (Schnakers et al., 2010). The first version of the NCS was based on pre-existing pain scales developed for non-communicative patients with advanced dementia and newborns. It consisted of four sub-scales assessing motor, verbal and visual responses to noxious stimuli as well as facial expression. Its total score ranged from 0 to 12. A second study including 64 patients with disorders of consciousness was performed in order to compare NCS scores observed at rest, in response to a non-noxious stimulus (i.e., tap on the shoulders) and in response to a noxious stimulus (i.e., nail bed pressure). Results showed that NCS total scores as well as motor, verbal and facial sub-scores were significantly higher in response to a noxious stimulus than to a non-noxious stimulus, reflecting the good sensitivity of the scale. However, no difference could be observed between noxious and non-noxious conditions for the visual sub-scores, suggesting that this sub-scale was not specific to nociception. The authors, therefore, decided to propose a new version excluding the visual sub-scale, the Nociception Coma Scale–Revised (Chatelle et al., 2012). Based on the revised version, a cut-off score of 4 has been defined as a potential clinical threshold for detecting pain in patients with disorders of consciousness. Chatelle et al. (2014a), using a 18-Fluoro-deoxyglucose PET scan, identified a significant positive correlation between NCS-R total scores and metabolism in the posterior part of the anterior cingulated cortex. Those results suggest that the NCS-R may constitute an appropriate behavioral tool to assess, monitor and treat nociception and pain in non-communicative patients with DOC (Chatelle et al., 2014b).

Nowadays, electroencephalographic (EEG) responses elicited by nociceptive laser heat pulses that selectively excite nociceptive free nerve endings in the epidermis, are widely adopted to investigate the peripheral and central processing of nociceptive sensory input (Bromm and Treede, 1984). The larger part of the laser-evoked potential (LEP) response is represented by a negative-positive biphasic wave (N2–P2), peaking 200–350 ms after hand stimulation and maximal at the scalp vertex and largely reflecting the activity of the bilateral operculo-insular and anterior cingulate cortex (Garcia-Larrea et al., 2003). The strong relationship between the N2 and P2 amplitudes in LEPs and the intensity of pain perception have been well documented (Kakigi et al., 1989; Bromm and Treede, 1991; Garcia-Larrea et al., 1997; Iannetti et al., 2005), and the correlations between N2 and P2 latencies and the intensity of pain perception were also reported (Iannetti et al., 2005). All these findings suggested a practice-oriented EEG-based pain prediction strategy, which adopted single-trial analysis to estimate LEP features for an effective pain prediction (Huang et al., 2013). Although widely used to investigate the function of nociceptive pathways in health and disease (Haanpaa et al., 2011), the physiological meaning of LEPs is still debated. Indeed, recent experimental evidence indicates that LEPs may reflect stimulus-triggered brain processes largely unspecific for nociception. As a matter of fact, non-nociceptive somatosensory stimuli (Garcia-Larrea et al., 1995), auditory stimuli (Picton et al., 1999), and even visual stimuli (Vogel and Luck, 2000) may all elicit a large “vertex potential” whose shape, scalp topography, and sensitivity resemble that evoked by nociceptive laser stimuli. It has been suggested that affective awareness is simpler and can persist despite very severe brain damage that is incompatible with conscious cognitive functions (Panksepp, 2005). In persons with disorders of consciousness, the pain matrix responds not only to direct nociceptive stimulation but also to complex stimuli that indicate the pain and suffering of another person (Yu et al., 2013). In a recent study, we showed the presence of a reliable cortical response after painful laser stimulation in both VS and MCS cases (de Tommaso et al., 2013), which co-existed with a substantial preservation of auditory Mismatch Negativity and absence of late cortical potentials evoked by somatosensory not noxious stimulus. In that study, we hypothesized that the cortical awareness toward pain stimulus may be a basal function for survival in state of vegetative autonomy, despite the absence of evident motor reaction to nociceptive inputs. A stimulation paradigm including nociceptive as well as auditory, visual and somatosensory evoked responses, may clarify the presence of cortical arousal toward these stimuli as measured by their saliency (Mouraux and Iannetti, 2009; Ronga et al., 2013). Our hypothesis is that in serious brain damage the capacity to orient the attention toward a relevant stimulus is generically preserved, with priority for painful inputs given their potentially dangerous consequences against the body. Moreover the cortical arousal toward relevant nociceptive and not nociceptive stimuli may be related to the degree of consciousness impairment, as measured by the Coma Recovery Scale-Revised (CRS-R) (Lombardi et al., 2007), and to the ability to organize a motor behavior against pain, as expressed by Nociception Coma Scale-Revised (NCS-R) (Chatelle et al., 2012). The aim of the present study was to examine the cortical response to series of visual, acoustic, somatosensory electric non-nociceptive and nociceptive laser stimuli, by means of the stimulation paradigm employed by Mouraux and Iannetti (2009), and to correlate event related potential features with the degree of consciousness impairment, as measured by Coma Recovery Scale-Revised (CRS-R), and with behavioral responding to pain, as measured by the revised version of Nociception Coma Scale (NCS-R).

## Cases

Nine patients were studied with severe acquired brain injury and disorders of consciousness (six males, three females, 44–76 years, mean 60.6 years) and 11 age- and sex-matched healthy volunteers as controls (seven males and four females, 50–68 years, mean 60.2 years; ANOVA test vs. patients age: *F* = 1.23, ns). Controls were selected among the patients' friends and family members, on the basis of absence of objective signs or history for general medical, neurological or psychiatric diseases, psychoactive drug use in the last 3 months, analgesics use in the last 48 h. Informed consent for the study was obtained from the legal representatives of all cases. The study was approved by the Ethics Committee of the Bari Policlinic General Hospital and the study procedure was in accord with the Declaration of Helsinki.

The brain injury was due to traumatic causes in four patients, intracranial hemorrhage in two patients, total anterior circulation infarct (TACI) in one patient and post-anoxic encephalopathy after cardiac arrest in two cases (Table 1). Level of consciousness was defined using repetitive standardized clinical evaluation with the Italian version of the Coma Recovery Scale-Revised (CRS-R) (Lombardi et al., 2007). The CRS-R is a unique tool, which includes the current diagnostic criteria for coma, vegetative state, and MCS, and allows the patient to be assigned to the most appropriate diagnostic category (Giacino et al., 2004). The CRS-R consists of 29 hierarchically organized items divided into 6 subscales addressing auditory, visual, motor, oromotor, communication, and arousal processes. Scoring is based on the presence or absence of specific behavioral responses to sensory stimuli administered in a standardized manner. The lowest item on each subscale represents reflexive activity, whereas the highest items represent cognitively mediated behaviors. The total score ranges between 0, the worst, and 23, the best. Of nine patients, five were in the vegetative state (patients 1–5), three were in minimally conscious state (patients 6–8) and one was emerged from a minimally conscious state (9).

**Table 1:** **Clinical, anamnestic and MRI features in the nine patients with disorder of consciousness**.

	**Patient 1**	**Patient 2**	**Patient 3**	**Patient 4**	**Patient 5**	**Patient 6**	**Patient 7**	**Patient 8**	**Patient 9**
Age	62	54	44	52	72	60	76	74	54
Etiology	Intracranial Haemorraghe	TBI	TBI	Anoxic encephalopathy	Anoxic encephalopathy	TBI	TACI	Intracranial Haemorraghe	TBI
Time from coma to ERPs	4 months	3 months	4 years	3 months	4 months	3 months	4 months	4 months	9 months
MRI lesions	Brain stem and left thalamus gliosis, diffuse subcortical leucoencephalopathy	Right frontal and bitemporal atrophy and gliosis	Left temporoparietal, periventricular, subcortical bifrontal and brain stem gliosis	Diffuse bihemispheric cortical necrosis with cerebral edema and lateral ventricular compression	Diffuse bihemispheric cortical and subcortical necrosis prevalent in brain stem and periventricular regions	Diffuse periventricular atrophy and brain stem hemorraghic lesions	Corpus callosum, left frontoparietal and paraventricular and right frontal ischemic lesions	Diffuse axonal lesions in right temporal, periventricular, corpus callosum and mesencephalic regions	Left hemispheric diffuse subcortical leucoencephalopathy and bifrontal gliosis
Level of consciousness	VS	VS	VS	VS	VS	MCS	MCS	MCS	MCS (emergence)
CRS-R	7	5	8	4	4	9	11	13	15
Auditory function	Auditory startle	Auditory startle	Localization to sound	Auditory startle	Auditory startle	Localization to sound	Localization to sound	Localization to sound	Consistent movement to command
Visual function	Visual startle	None	Visual startle	None	None	Fixation	Visual pursuit	Visual pursuit	Visual pursuit
Motor function	Flexion withdrawal	Abnormal posturing	Flexion withdrawal	Abnormal posturing	Abnormal posturing	Flexion withdrawal	Flexion withdrawal	Automatic motor response	Localization to noxious stimulation
Oromotor/Verbal function	Oral reflexive movements	Oral reflexive movements	Oral reflexive movements	Oral reflexive movements	None	Oral reflexive movements	Oral reflexive movements	Oral reflexive movements	Oral reflexive movements
Communication	None	None	None	None	None	None	Non-functional: Intentional	None	Functional: Accurate
Arousal function	Eye opening w/o stimulation	Eye opening w/o stimulation	Eye opening w/o stimulation	Eye opening with stimulation	Eye opening w/o stimulation	Eye opening w/o stimulation	Eye opening w/o stimulation	Eye opening w/o stimulation	Eye opening w/o stimulation
Muscle-tendon retraction or HO	Present	Present	Present	None	Present	None	Present	None	Present
Neuromotor condition	Spastic tetraplegia	Spastic tetraplegia	Spastic tetraplegia	No spastic tetraplegia	Spastic tetraplegia	No spastic tetraparesis	Spastic tetraplegia	Spastic hemiplegia	Spastic tetraparesis
Pressure ulcer	Present	Absent	Present	Absent	Present	Absent	Absent	Absent	Present
NCS-R	4	1	6	4	3	6	4	7	5
Motor response	Flexion withdrawal	Abnormal posturing	Flexion withdrawal	Abnormal posturing	Abnormal posturing	Localization to noxious stimulation	Flexion withdrawal	Localization to noxious stimulation	Flexion withdrawal
Verbal response	None	None	Groaning	Groaning	Groaning	Groaning	None	Groaning	Groaning
Facial expression	Grimace	None	Grimace	Oral reflexive movement	Grimace	Grimace	Grimace	Cry	Grimace

*TBI, traumatic brain injury; TACI, Total Anterior Circulation Infarct; VS, Vegetative State; MCS, Minimal Conscious State*.

All patients were evaluated at least 3 months after the acute event. The level of consciousness was assessed with CRS-R 1 week before the neurophysiological evaluation, the day of event-related potentials testing and 1 week after this testing. Most of the patients presented with muscle and tendon retractions, heterotopic ossifications, pressure ulcers and spasticity, which are considered potential triggers of chronic pain (see Table 1). The NCS-R involved fingernail pressure as the noxious stimulus for a minimum of 5 s, stopping the press as soon as a behavioral response was observed. Behavioral responses were recorded for 10 s after each noxious stimulus. Patients were assessed the day of event-related potentials testing. Part of neurophysiological findings obtained with seven patients was reported in de Tommaso et al. (2013).

## Method

### Recording

In addition to the 19 standard positions of the international 10–20 system, 45 additional electrodes were placed on the x, y, and z co-ordinates provided by the Advanced Source Analysis (ASA) software (ASA version 4.8.1; ANT Software, Enschede, The Netherlands; http://www.ant-neuro.com). The reference electrode was placed on the nose, the ground electrode was in Fpz and one electrode was placed above the right eyebrow for electro-oculogram (EOG) recording. Impedance was kept at 10 kor less. The EEG and EOG signals were amplified with a bandpass of 0.5–80 Hz, digitized at 250 Hz and stored on a biopotential analyser (Micromed System Plus; Micromed, Mogliano Veneto, Italy; http://www.micromed-it.com).

Stimulation procedures. Two series of 25 laser, electrical, auditory and visual stimuli were presented in random order, at an inter-stimulus interval of 10–12 s. The laser stimulus was applied on the dorsum of the right hand with the random use of two series of 25 CO_2_ laser stimuli, settled at a fixed duration of 30 ms and an intensity of 9 W. These stimulus parameters were applied to control subjects before the stimulating session for at least 10 times and a verbal analogical scale from 0 (no pain) to 10 (maximum tolerable pain- 4 pain thresholds as pinprick sensation). These laser stimulation parameters caused a pain sensation varying from 5 to 7 levels in all control subjects. Electric stimulations of 200 μs duration and 30 mA were employed in controls, determining a clear non-painful mechanical stimulation. The dorsum of the right hand was also stimulated by the means of felt tip electrodes in patients and controls, according to the previously reported procedure (de Tommaso et al., 2013). Auditory stimulation was done by means of 1500 Hz with an intensity of 70 dB SPL and duration of 75 ms (rise and fall time 10 ms) pure tones, delivered binaurally through insert earphones. Visual stimulation was delivered by goggles worn by patients during the entire task. A black-white checkerboard with 98% (high) contrast, 0.5 c/d spatial frequency and 1-s duration appeared on a black background.

### Data analysis

All recordings were analyzed by the first author, who was blinded to the patients' VS or MCS diagnosis. Trials contaminated by ocular or muscle artifacts were excluded from the analysis, after a visual inspection and individuation of artifacts features, as amplitude, phase and frequency. An automatic artifact-rejection system excluded all the EEG tracks including activities with the above reported artifact characteristics, taking also into consideration the signals features reported on the EOG channels. The averaging procedure was conducted on at least 10 artifact—free tracks following the laser, electrical, visual and auditory stimuli, with 100 ms prestimulus and 1 s post-stimulus time. Laser (LEPs), late somatosensory (LSEPs), auditory (LAEPs) and visual (LVEPs) evoked responses were identified as N2-P2 vertex complex in patients and controls, according to Valeriani et al. (1996) and Mouraux and Iannetti (2009).

The main event-related components' amplitude and latency values were computed by means of an automatic wave scoring program according to ASA software, after the visual inspection of the laser, electric, acoustic and visual N2 and P2, according to Valeriani et al. (1996) and Mouraux and Iannetti (2009). The program calculated the maximum negativity and positivity in the 150–450 ms correspondent times, after a baseline correction and the subtraction of the pre-stimulus signal. The automatic detection of a negative-positive amplitude variation of at least 2 uV in respect to corrected baseline on at least 10 channels (including at least 2 derivations among the central-parietal ones) was used to confirm the presence of a reliable response, confirmed by the EEG visual inspection. The same automatic detection was run out in the time interval 70–150 ms to ensure the presence of a reliable N1 and P100 response following respectively the auditory and visual stimuli on at least two temporo-parietal and parietal-occipital derivations. The early somatosensory evoked potentials were also recorded in another laboratory (Cruccu et al., 2008), in five out of nine patients, stimulating the right and left median nerve. They were compared with those recorded in a group of 30 healthy age and sex-matched controls. The grand average in controls and the event related potentials responses in single patients, were topographically represented on amplitude maps provided by ASA software.

### Statistical analysis

In consideration of the small patients' groups, a Student *t*-test was employed to compare individual latency and amplitude values to control group. For this analysis, data detected on Cz derivation were included, which represented a reliable channel in patients with a detectable response, according to the above reported automatic and visual inspection. A One-Way ANOVA analysis with *post-hoc* Bonferroni test was designed for event-related potentials (ERP) features, considering the groups as factor. In order to find the ERP features that best separated among VS, MCS and controls, a stepwise discriminant analysis was run, using Mahalanobis distance, F probability of 0.05 for entry and 0.1 for removal. classification function coefficient by means of Fisher's linear discriminant test, and leaving one out of final classification. ERP features were also correlated with CRS-R and NCS-R scales by means of Pearson correlation test. In both correlation and discriminant analyses, the absent ERP were represented with 0 as amplitude value. For statistical analysis, the SPSS version 21 was employed.

## Results

**Figure 1:**
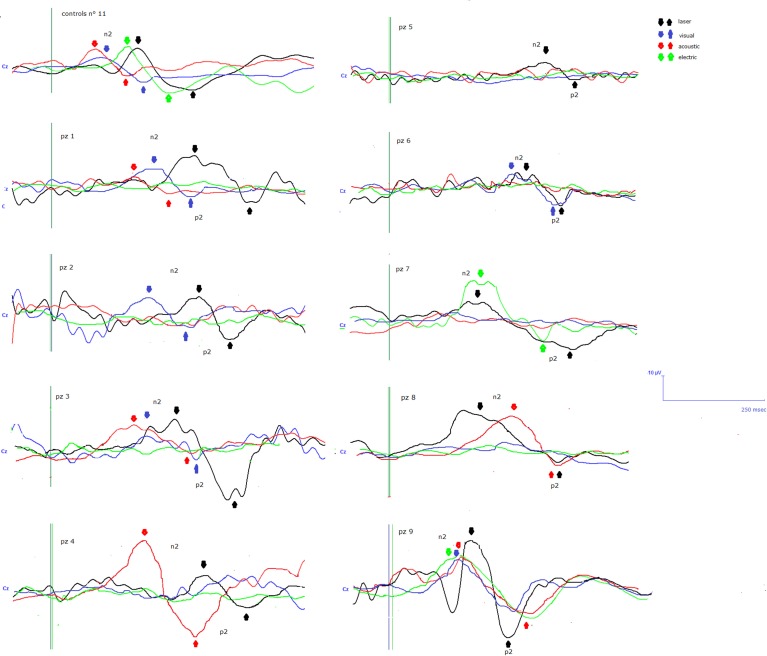
**N2-P2 vertex complexes by Cz (referred to nasion) derivation, obtained in the 9 patients, numbered in accord with Table 1**. For each case, the average of at least 10 artifact—free response by auditory, visual, somatosensory electric and laser stimuli in the time range 0–500 ms was reported, when visual and automatic scoring confirmed the presence of a reliable response. At the top of the figure the grand averages across normal controls are also represented.

**Figure 2:**
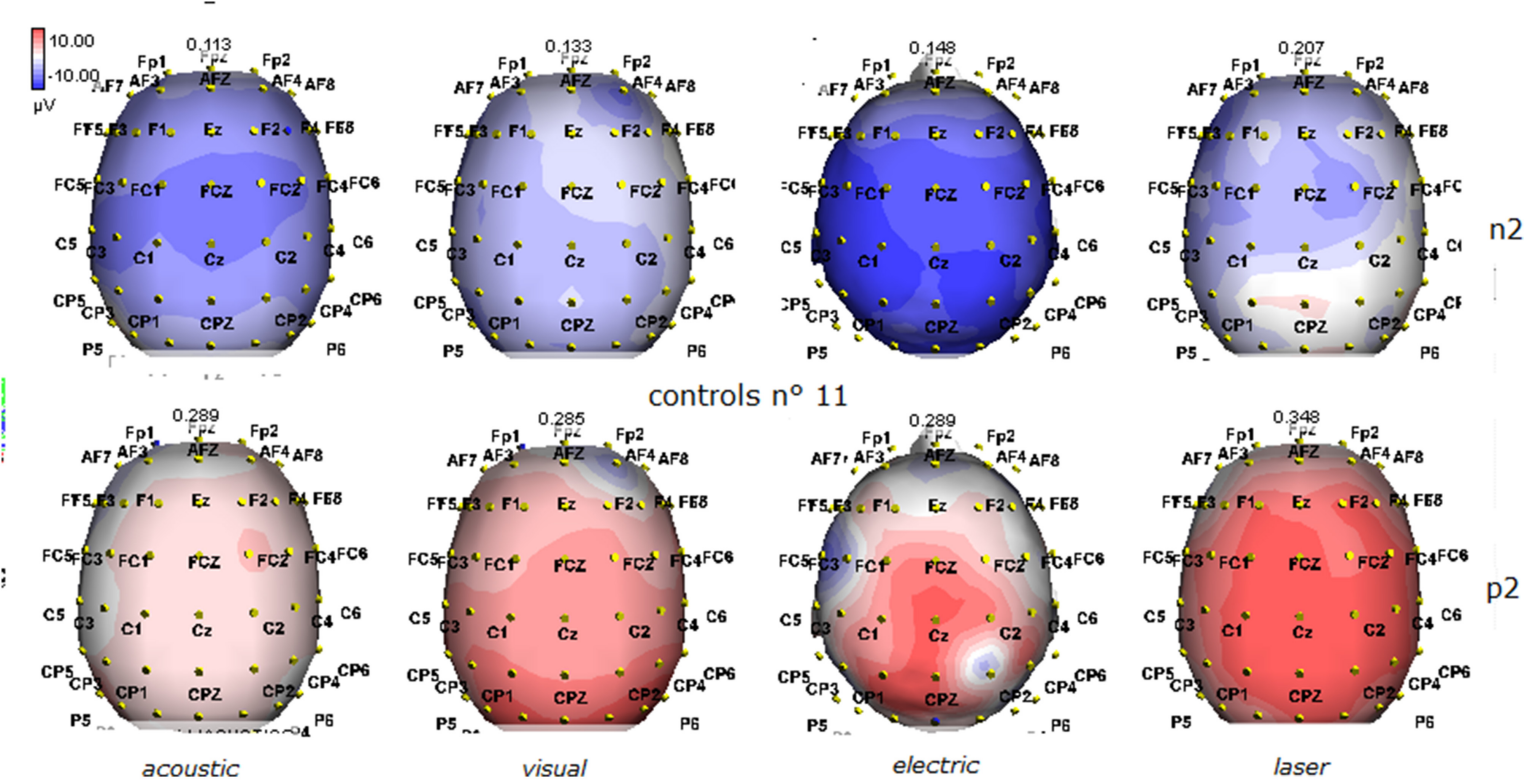
**Grand average maps of N2 and P2 amplitudes by, acoustic, visual, electric and laser stimuli in 11 controls are reported**. Maps were constructed according to ASA ANT software vers. 8.4.

**Figure 3:**
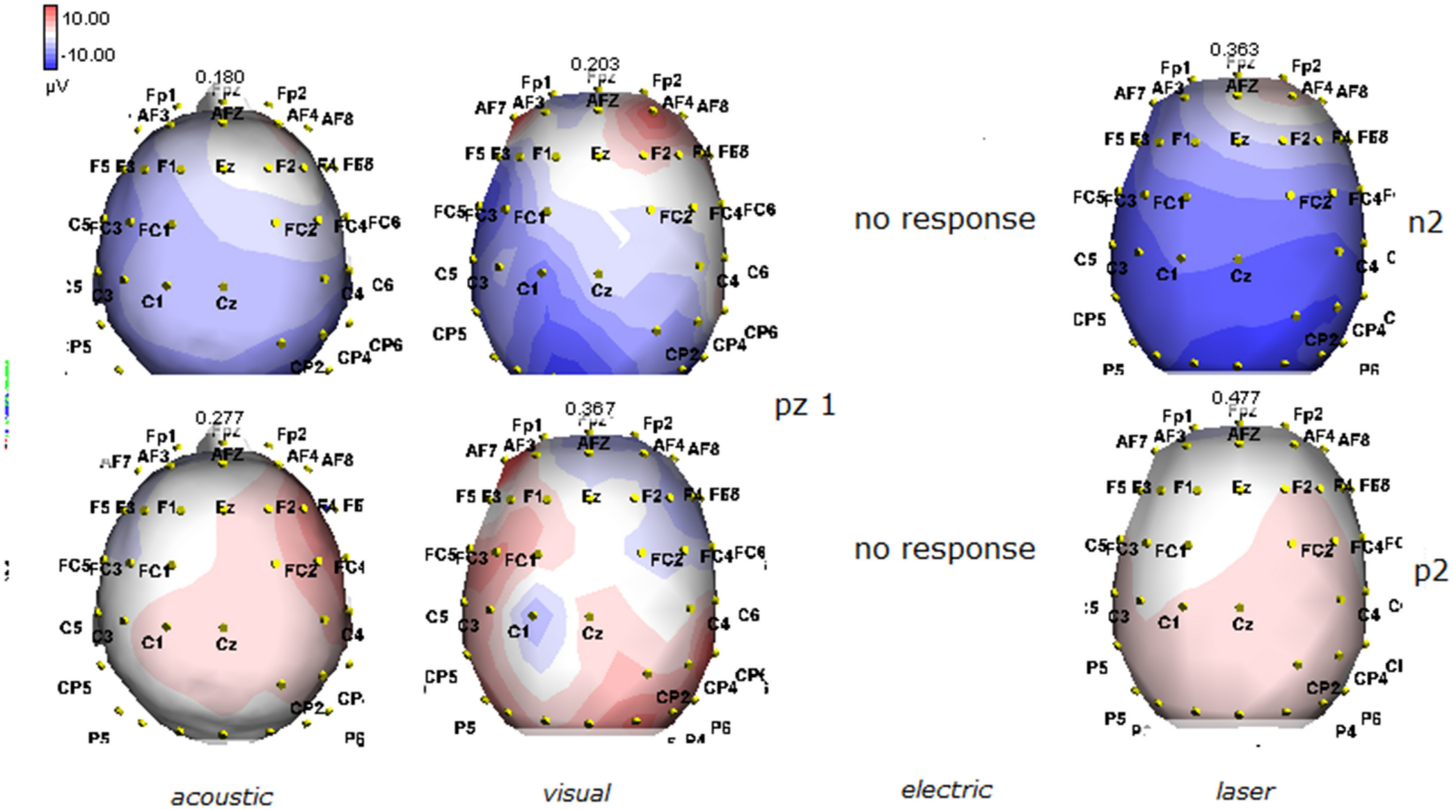
**Topographic maps of N2 and P2 waves amplitudes in patient 1 (VS-see Table 1)**. Maps were constructed according to ASA ANT software vers. 8.4, when visual and automatic scoring confirmed the presence of a reliable response. In this patient, all but electric stimuli elicited a reliable response.

**Figure 4:**
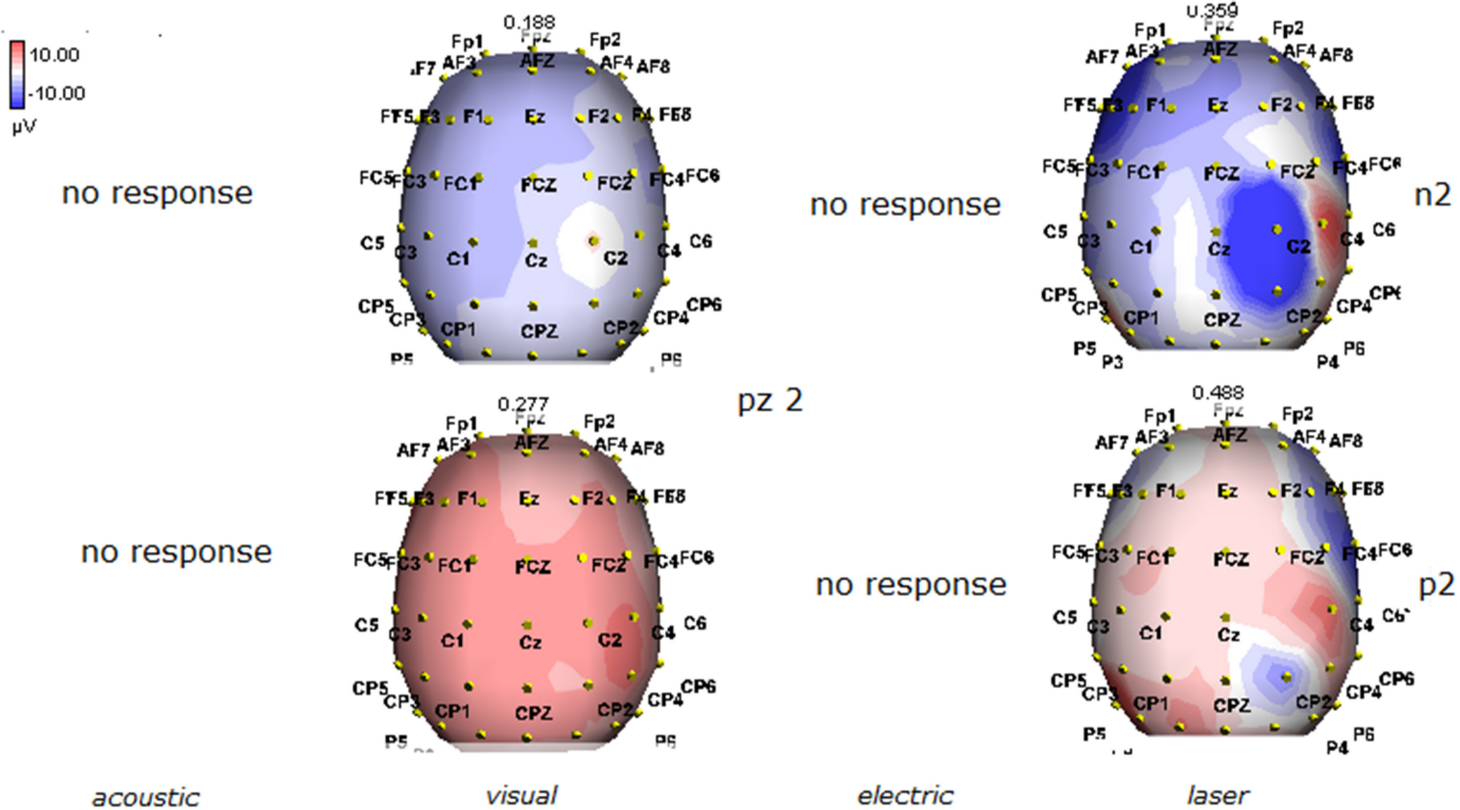
**Topographic maps of N2 and P2 waves amplitudes in patient 2 (VS-see Table 1)**. In this patient, only visual and laser stimuli elicited a reliable response, with a prevalent representation of N2 and P2 waves by laser stimuli on the right central derivations.

**Figure 5:**
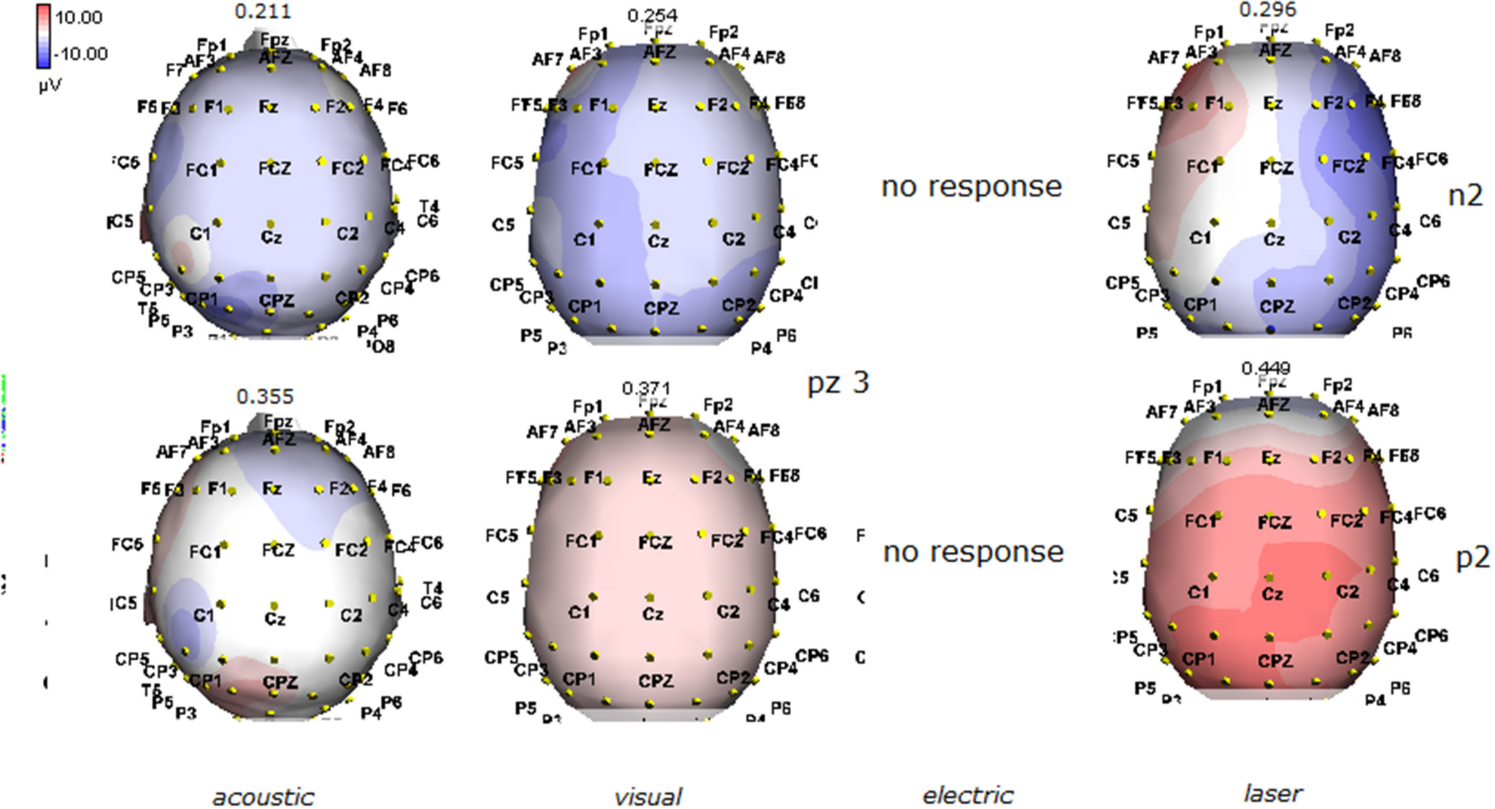
**Topographic maps of N2 and P2 waves amplitudes in patient 3 (VS-see Table 1)**. This patient did not show a reliable cortical response under electric stimuli, while laser stimuli evoked a negative component with prevalent representation over the right hemisphere.

**Figure 6:**
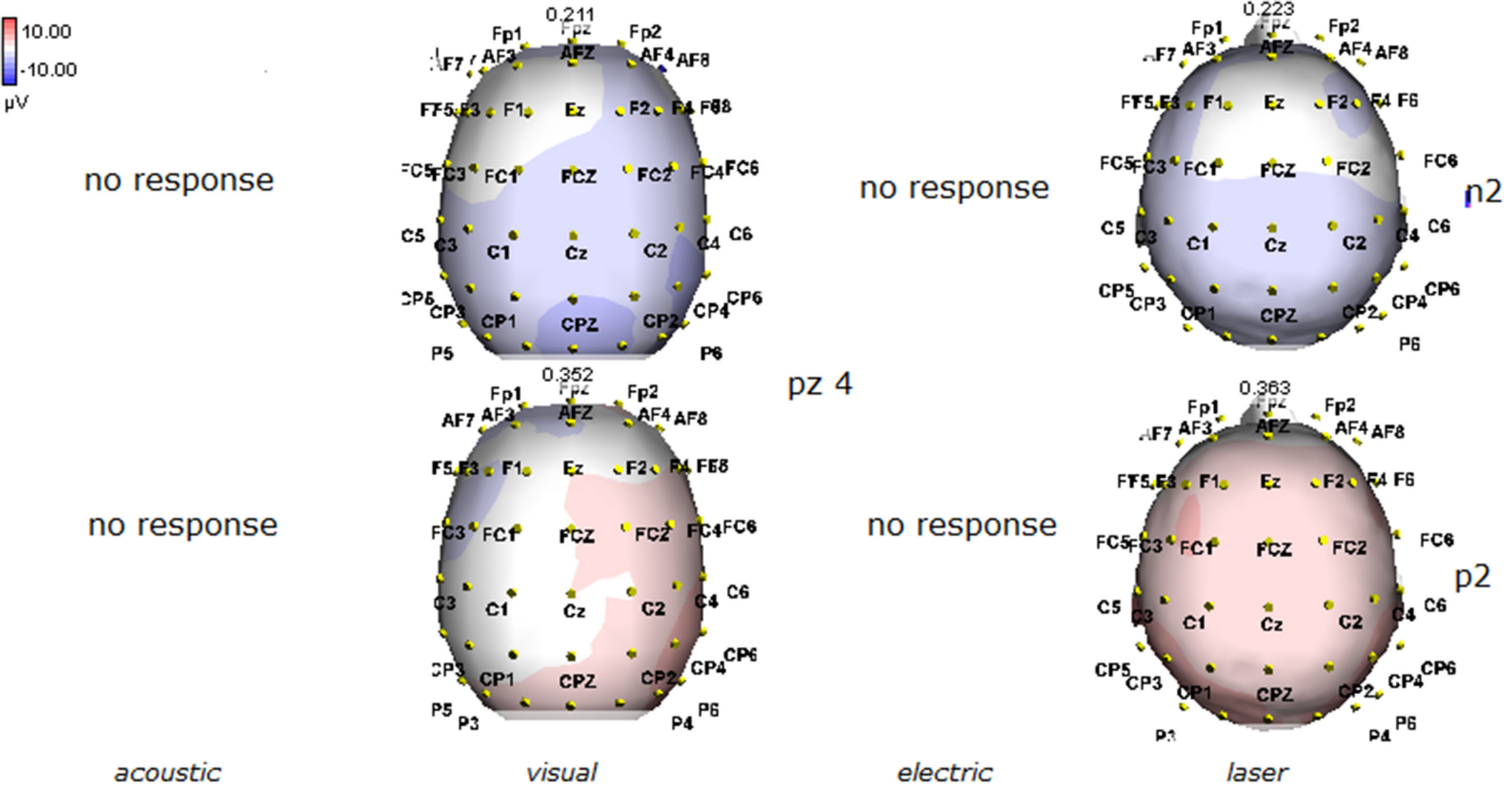
**Topographic maps of N2 and P2 waves amplitudes in patient 4 (VS-see Table 1)**. A part from event related potentials by laser stimulation, low amplitude N2P2 complex by visual stimuli was detectable.

**Figure 7:**
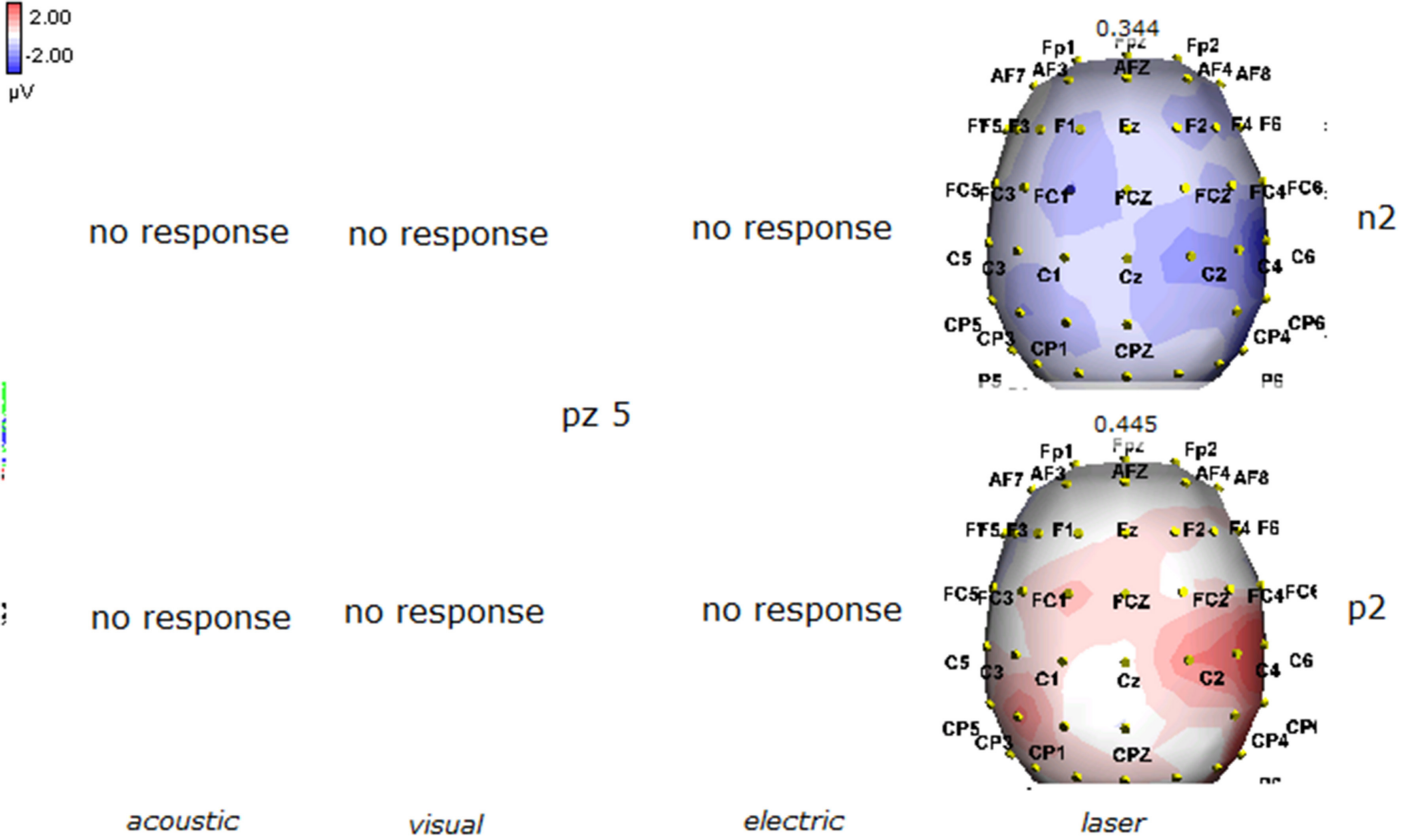
**Topographic maps of N2 and P2 waves amplitudes in patient 5 (VS-see Table 1)**. In this patient, only low voltage N2P2 waves by laser stimuli were detectable, with a reduced representation over the midline.

**Figure 8:**
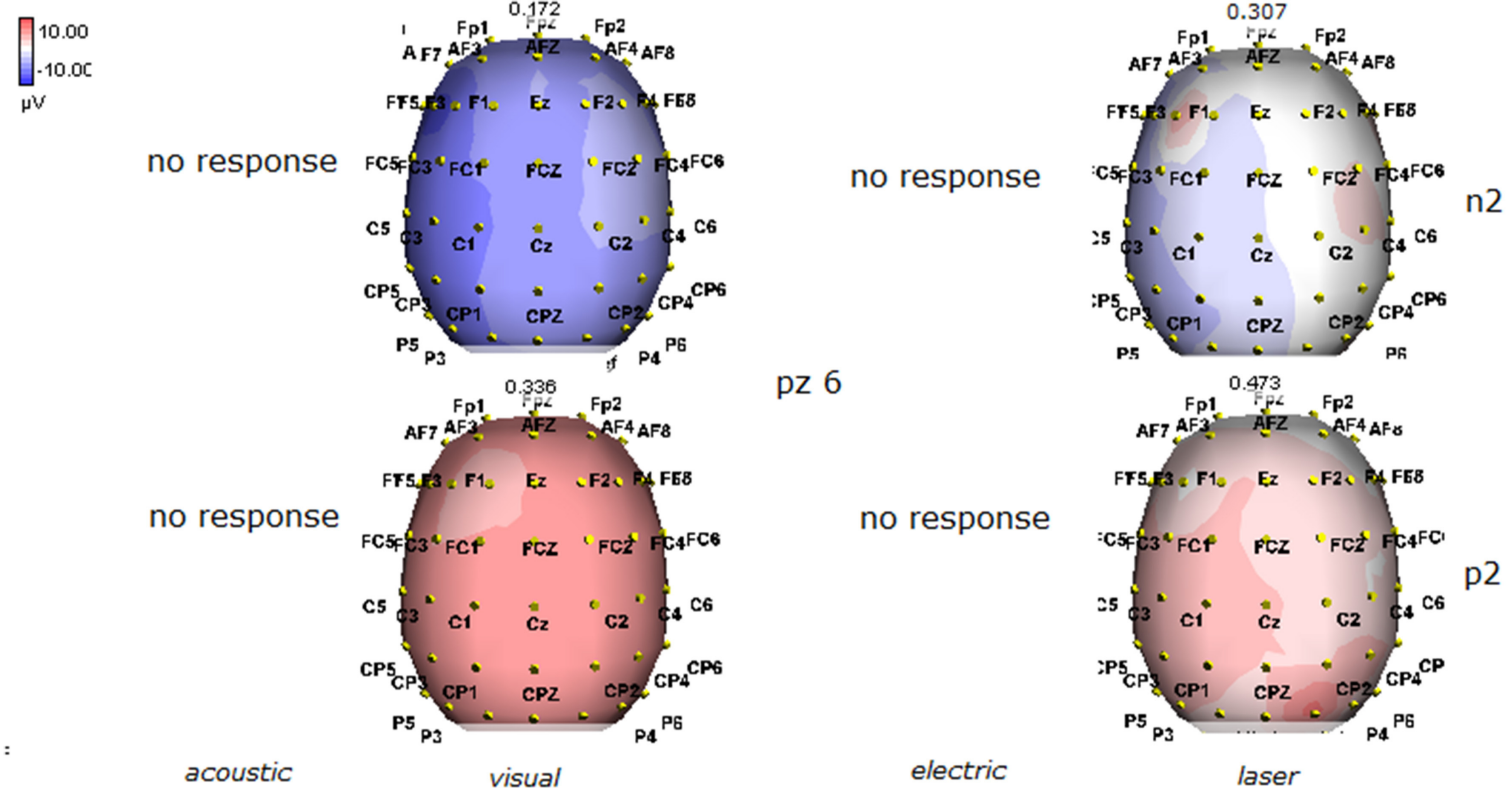
**Topographic maps of N2 and P2 waves amplitudes in patient 6 (MCS-see Table 1)**. In this patient, visual and laser stimuli elicited a reliable response.

**Figure 9:**
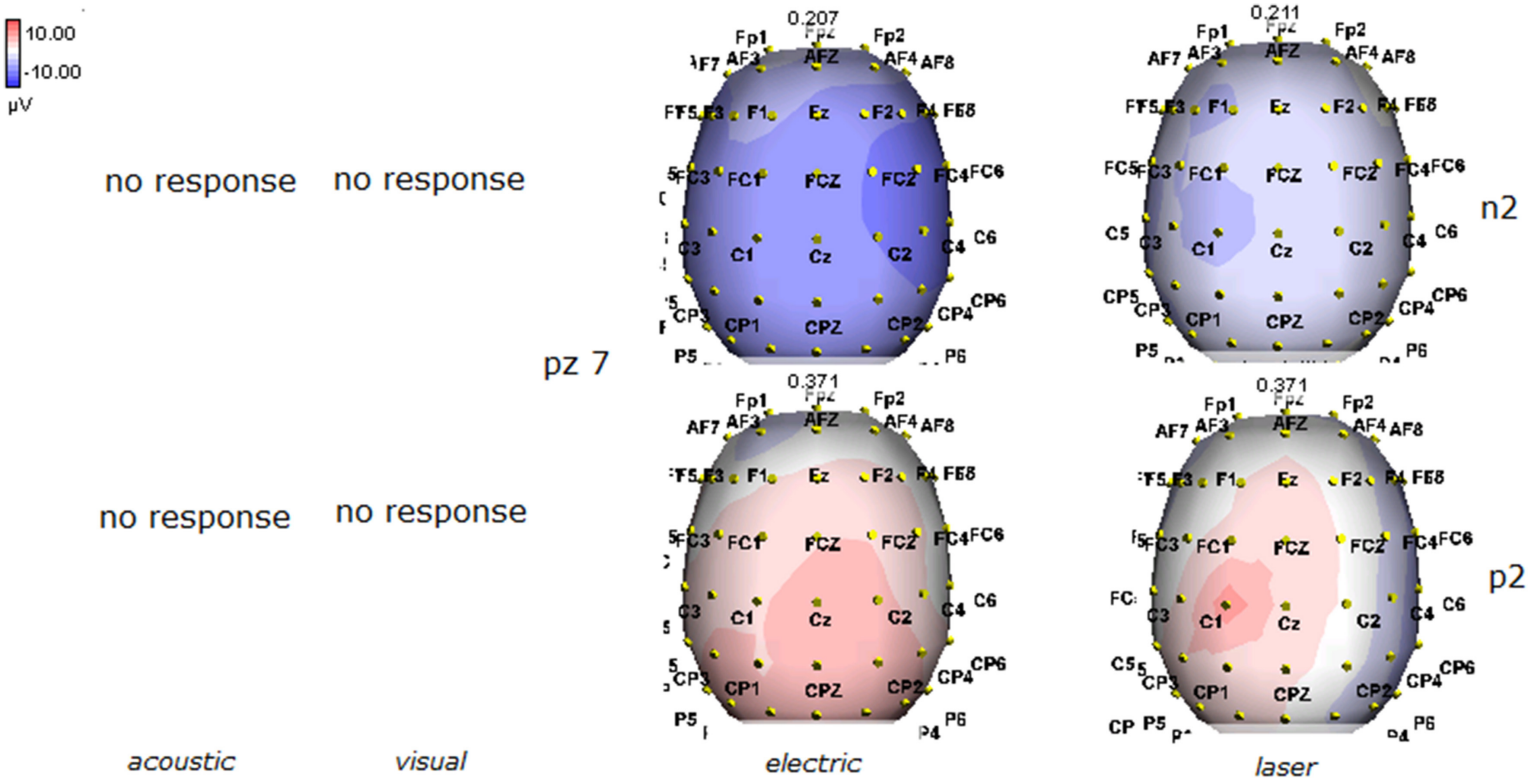
**Topographic maps of N2 and P2 waves amplitudes in patient 7 (MCS-see Table 1), who showed cortical negative positive complex under electric and laser stimuli**.

**Figure 10:**
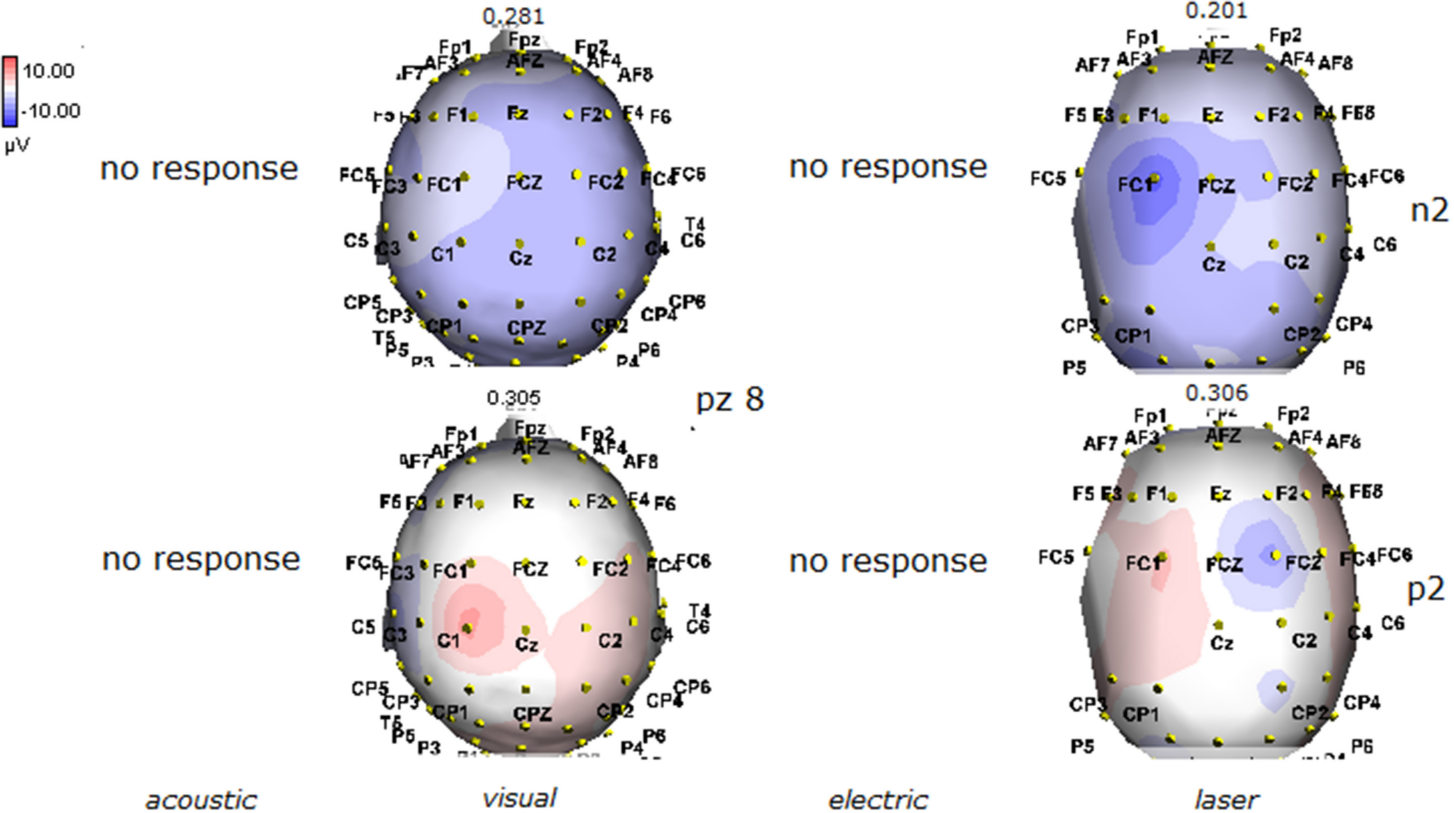
**Topographic maps of N2 and P2 waves amplitudes in patient 8 (MCS-see Table 1)**. This patient showed a clear negative response under visual stimuli, with a visual P2 mainly represented over the right temporo-parietal regions. A prevalent frontal distribution was detectable for laser evoked responses.

**Figure 11:**
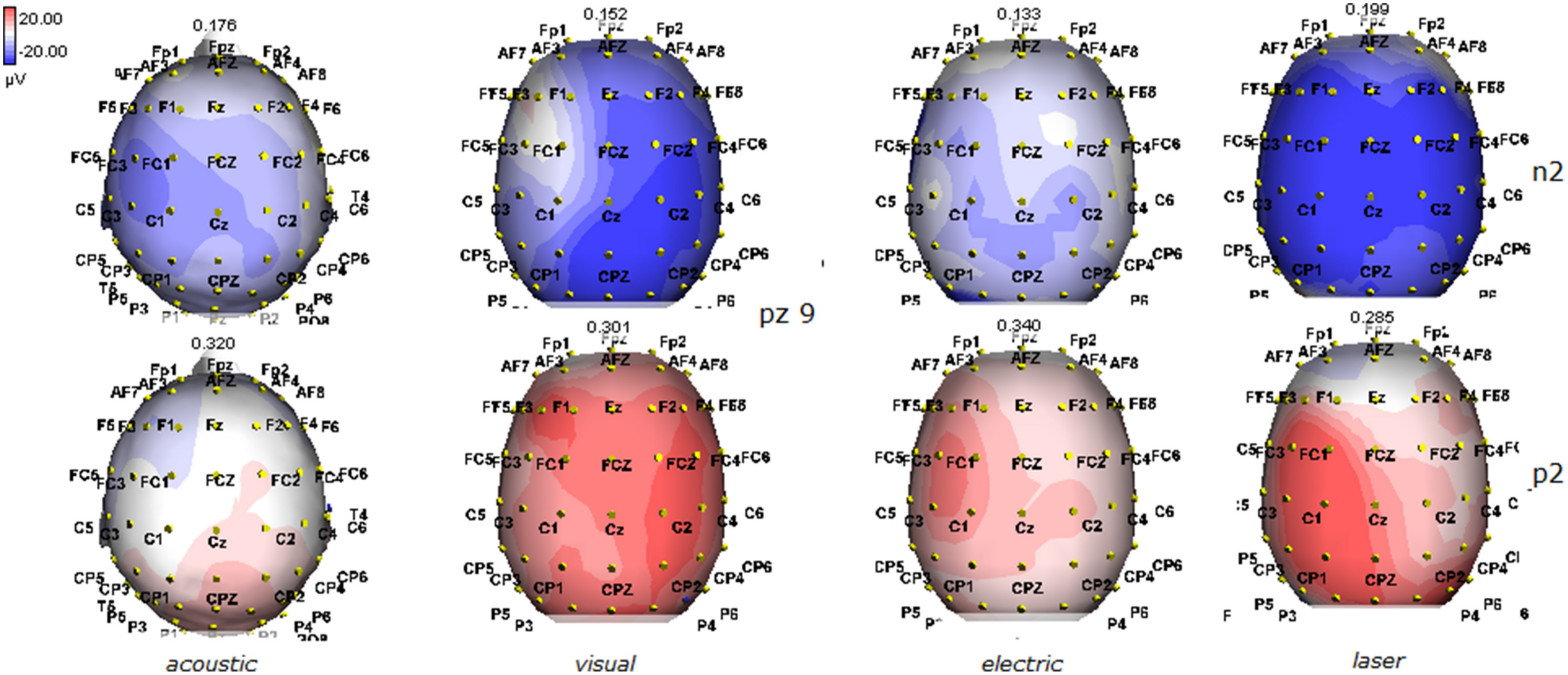
**Topographic maps of N2 and P2 waves amplitudes in patient 9 (MCS-see Table 1)**. This patient showed a clear response to all the stimulation modalities, with large N2P2 complex under visual, electric and laser stimuli. These latter stimulation elicited a P2 response with prevalent distribution over the left hemisphere.

Details regarding the nine cases involved in the study are reported in Table 1. One patient (n° 9) was emerging from a MCS. The NCS-R total score obtained in MCS patients (5.50 ± 1.29) was not significantly higher than the score of VS patients (3.6 ± 1.81; Student' s *t*-test 1.75 n.s.). Regarding the ERP, controls showed a negative positive vertex complex related to all stimulation modalities. (Figure 1; Table 2). A similar scalp distribution emerged for the N2P2 evoked by different stimuli, with a prevalent posterior shift for the visual event related N2 and P2 responses, and a reduced amplitude for the waves obtained by the acoustic stimuli (Figure 2). In all patients, early cortical responses from auditory and visual stimuli were recognizable in line with the visual and automatic detection score. In addition, all patients submitted to early somatosensory evoked responses showed a recognizable N20. Four of the 5 VS patients presented with normal amplitude LEPs, with a clear N2 and P2 latency prolongation. The figures, showing single patients averaging, indicate the presence of LEPs in all cases, with visual event related potentials in cases 1, 2, and 3 and acoustic related potentials in cases 1, 3, and 4. In case 5, the automatic wave recognition confirmed the presence of low voltage LEPs. (Table 2; Figures 1, 3–7). Two patients with MCS presented with prolonged LEPs, in 2 cases the N2 and P2 latencies were within normal limits, in two patients late somatosensory evoked potentials were evident (cases 7 and 9). Late visual and the late acoustic potentials were detectable in cases 6 and 8, respectively. Only in case 9, who was emerging from a MCS, all the stimulation modalities evoked a reliable vertex response (Table 2; Figures 1, 8–11).

**Table 2:** **Latencies and amplitudes of vertex N2-P2 components in patients with disorder of consciousness**.

	**Patient 1**	**Patient 2**	**Patient 3**	**Patient 4**	**Patient 5**	**Patient 6**	**Patient 7**	**Patient 8**	**Patient 9**	**Controls M SD**
**L-AEPs**										
P2	277^***^	Absent	355^***^	336^***^	Absent	Absent	Absent	305^***^	320^***^	216
latency										11
N2	180^***^	Absent	211^***^	172^**^	Absent	Absent	Absent	281^***^	176^***^	118
latency										12
N2-P2 amplitude	4,78^***^	0	11.9	42^***^	0	0	0	13	19	10.7
										6.4
**L-VEPs**										
P2	277^***^	267^***^	375^***^	Absent	Absent	352^***^	Absent	Absent	301^***^	215
Latency										26
N2	367^***^	188^***^	250^***^	Absent	Absent	211^***^	Absent	Absent	152	152
latency										10
N2-P2 amplitude	10.70	10.37	7.9	0	0	11	0	0	19	10.4
										4.4
**l-SEPs**										
P2	Absent	Absent	Absent	Absent	Absent	Absent	361^***^	Absent	320^***^	280
latency										11.1
N2	Absent	Absent	Absent	Absent	Absent	Absent	207^***^	Absent	133	138
latency										14.1
N2-P2 amplitude	0	0	0	0	0	0	23	0	23,9	15
										5.5
**LEPs**										
P2	453^***^	469^***^	446^***^	463^***^	445^***^	363^*^	441^***^	309	285	341
latency										14.1
N2	377^***^	363^***^	246^*^	−367^***^	344^***^	213	−211	201	188	210
latency										15.5
N2-P2 amplitude	19	20	27.44	8.11	4^***^	12.4	17	22	32	15.5
										6.5

In the last column, mean and standard deviation values from 11 normal controls are reported. The results of Student's t-test for individual comparison with control groups are also reported

* *p < 0.05*;

** *p < 0.01*;

*** *p < 0.001*.

Considering that the auditory, somatosensory and visual N2 and P2 waves were not recognizable in many cases, the ANOVA and discriminant analysis among groups was not possible for these event related potentials. The laser N2 and P2 latency was significantly different among groups, for a significant increase in VS cases compared to the control group (laser N2 ANOVA *F* = 6.47 DF 2 p 0.008; laser P2 *F* = 8.35 DF 2 p 0.003; Bonferroni test VS patients vs. controls for N2 and P2 latencies: p < 0.01). The laser N2-P2 amplitude was not correlated with the CRS-R and NCS-R scores, while N2 and P2 latencies prolongation corresponded to reduced consciousness recovery signs (Pearson test: CRF vs. laser N2 latency 0.6 p 0.041; CRF vs. laser P2 latency 0.63 p 0.033). We carried out a correlation analysis between visual, electric and auditory ERP amplitudes and clinical scores, attributing 0 value to absent potentials. The vertex complex amplitude by acoustic stimuli was significantly correlated with NCS-R scale and the electric event related response was correlated with CRS-R scale (Pearson test NCS-R vs. acoustic N2-P2: 0.62 p 0.035; CRF-R vs. electric N2-P2: 0.66 p 0.032).

**Figure 12:**
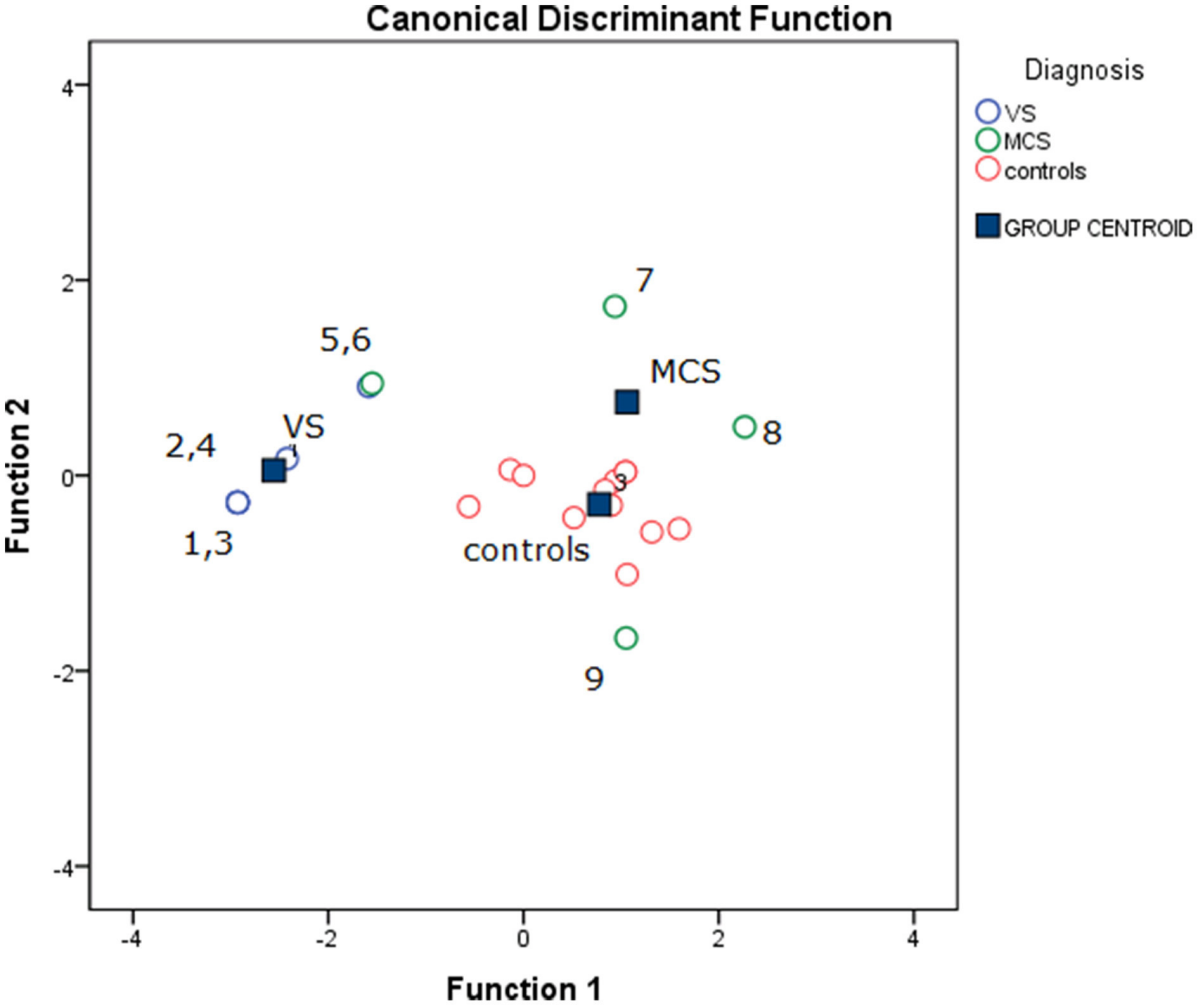
**Groups classification according to discriminant analysis, as showed in Table 3**. Three centroids are reported for VS (Vegetative State), MCS (Minimal Conscious State) and controls. Single cases numbers are reported, according to Table 1. The discriminant functions were based in order on acoustic, electrical and visual N2-P2 amplitudes, while LEPs amplitude was excluded by step-wise leave one out method. (see Table 3).

The stepwise discriminant analysis by leave one-out method selected, in order, auditory, electric and visual N2-P2 amplitudes as the best discriminating variables among VS, MCS and control groups, while LEPs features were excluded from the analysis. The canonical discriminant function, correctly classified 90% of the original grouped cases (100% in VS group) and 85% of the cross validated grouped cases (Table 3; Figure 12).

Table 3**Top Results of discriminant analysis by leave-one-out method**.

**Table:** 

**Step**		**Tollerance**	**F for removal**	**Min. D square**	**Among groups**
**INCLUDED VARIABLES**					
1	A-N2P2	1.000	0.043		
2	A-N2P2	0.761	0.006	0.356	MCS/C
	E-N2P2	0.761	0.000	0.593	VS/C
	V-N2P2	0.77	0.005	0.583	VS/MCS
**EXCLUDED VARIABLES**					
0	A-N2P2	1.000	0.043	0.593	VS/C
	V-N2P2	1.000	0.044	0.598	MCS/VS
	E-N2P2	1.000	0.001	0.356	MCS/C
	L-N2P2	1.000	0.700	0.038	VS/C
1	V-N2P2	0.997	0.001	1.089	VS/C
	E-N2P2	0.761	0.000	1.179	MCS/C
	L-N2P2	0.988	0.652	0.605	VS/MCS
2	L-N2P2	0.988	0.672	1.558	VS/MCS

**Table:** 

Diagnosis			Classification			Total
			**VS**	**MCS**	**Controls**	
Original		VS	5	0	0	5
		MCS	1	2	1	4
		C	0	0	11	11
	%	VS	100.0	0.0	0.0	100.0
		MCS	25.0	50.0	25.0	100.0
		C	0.0	0.0	100.0	100.0
Cross-validated		VS	5	0	0	5
		MCS	1	1	2	4
		C	0	0	11	11
	%	VS	100.0	0.0	0.0	100.0
		MCS	25.0	25.0	50.0	100.0
		C	0.0	0.0	100.0	100.0

*The acoustic (A-N2P2), visual (V-N2P2) and electric (E-N2P2) event related potentials were introduced in the analysis, while laser evoked potentials (L-N2P2) were excluded for the low discriminating power among groups*.

*At the bottom, classification function coefficients are reported on the basis of the selected variables*.

*VS, Vegetative State; MCS, Minimal Conscious State; C, controls*.

## Discussion

The stimulation and analysis paradigm useful to detect a cortical response toward salient stimuli in accord to Mouraux and Iannetti (2009), confirmed in our control group the presence of a vertex complex by the different types of stimuli. The negative-positive complex obtained from auditory, visual and somatosensory non-nociceptive stimuli resembled the morphology and topographic distribution of the laser N2-P2 waves, confirming that pain may activate the same cortical zones as other potentially relevant stimuli. In our severe brain damaged patients, a reliable LEP response was present also in patients in vegetative state, in accord with our recent study (de Tommaso et al., 2013). Amplitude of LEPs was reduced only in one vegetative state patient (i.e., resulting within normal limit in other VS and MCS cases). A significant N2 and P2 latency prolongation confirmed our previous study, in which a disturbance in cortical inter-connections was suggested as the cause of this abnormality (Kassubek et al., 2003; Bekinschtein and Manes, 2008; de Tommaso et al., 2013). In all but one patient, who was in an early MCS resolution, we found that the other stimulation modalities were not always able to evoke a reliable cortical response, so in some cases LEPs were associated to visual and/or acoustic response, and only in one case to late somatosensory non-nociceptive potentials. In the present evaluation, we gave further attention to artifact detection, employing an automatic rejection method which took into consideration the similarity with confounding factors as ocular, limbs and head movements. In addition, we used an automatic detection method supported by visual inspection, to ensure the presence of low voltage responses, as in case 5. In all patients the same method allowed us to exclude an early central visual and acoustic conduction failure by means of the detection of the auditory N1 and visual P100, while early somatosensory evoked potentials excluded peripheral and central somatosensory conduction failure in 6 patients, as reported in our previous study (de Tommaso et al., 2013). According to LEPs findings, also in the case of visual, auditory and electrical evoked responses, a P2 latency increase was present in almost all cases, with N2 prolongation in most of the patients, confirming cortical inter-connection failure and a functional re-arrangement enabling to preserve a cortical reaction. In most of the patients, the topographic distribution of cortical responses evoked by different stimulation modalities, did not resemble that observed in normal groups, probably for the presence of large cortical-subcortical lesions with an increased representation of event related responses on scalp zones corresponding to cortical compensatory areas.

The stimulation paradigm here employed is considered reliable in measuring the novelty and salience of different stimuli, expressing the activity of those cortical areas devoted to arousal and potential motor reaction (Mouraux and Iannetti, 2009; Ronga et al., 2013). The presence of LEPs in all cases, independently from the degree of consciousness impairment, confirmed our hypothesis that cortical arousal toward pain is a primary function useful to detect potentially dangerous factors also in absence of motor defense ability. Two previous studies reported an activation of the affective pain network (i.e., ACC and insula) in 30% of patients in a VS in response to noxious stimulation as well as pain cries (Markl et al., 2013; Yu et al., 2013). The last study, nevertheless, also showed, in parallel to previous findings, that the connectivity in the whole pain network was significantly decreased as compared to patients in a MCS (Kotchoubey et al., 2013). Also in our study, the mean time for pain stimuli cortical reaction, expressed by N2 and P2 latency, was increased in patients in vegetative state, confirming a severe impairment in functional inter-connections in such severe damaged patients, which on the other hand did not preclude the appearance of a cortical reaction. Even though this suggests an altered perception in patients in a VS, the activation of the affective pain network might denote the presence of residual pain perception. As a minority of patients behaviorally diagnosed as VS have previously shown brain activation in response to active cognitive tasks (Monti et al., 2010; Cruse et al., 2011), it is also plausible to assume that the cognitive residual functions are dedicated to the stimuli with potentially dangerous valence (Chatelle et al., 2014b). In fact, visual, acoustic and somatosensory not nociceptive stimuli were alternatively under the threshold of salience for both VS and MCS patients. In our study, group analysis consistency was limited by the small case series. With the caution imposed by the few cases, we could argue that the discriminant analysis results confirmed that LEPs presence may not account for a better consciousness preservation, rather the cortical arousal toward the other sensory stimuli (e.g., auditory, visual and somatosensory electric) may accurately discriminate VS patients from MCS and control. Interestingly, the MCS patient with early consciousness recovery was classified as normal. This patient (case 9) presented with cortical responses to all the delivered stimuli, confirming that the detection of relevance increases with the recovery of cognitive and motor abilities. Although the reliability of correlation between ERP features and clinical scores is to be taken with caution given the small series, one could underline the association between prolonged LEPs and low coma recovery signs. At the same time, behavioral response to pain, as expressed by R-NCS score, was associated with increased auditory vertex potentials, suggesting that motor reaction against noxious stimuli may be a better function of global cognitive improvement than pain awareness and perception.

In conclusion, this study confirmed that pain arousal may be a primary function also in severe brain damaged patients, who probably finalize all cortical arousal resources toward those stimuli which may be potentially dangerous for life maintenance. Moreover the relevance of other stimulus modality may be perceived when a better reorganization of cognitive and motor reactions is present. This underlines the importance of considering the potential experience of pain also in patients in vegetative state and to appropriately assess a possible treatment also in those patients.

## Author contributions

Marina de Tommaso; study design and coordination, manuscript preparation, data analysis. Jorge Navarro; study design, patients clinical assessment, manuscript preparation. Crocifissa Lazillotti: patients clinical assessment. Katia Ricci: EEG recording. Francesca Buonocunto: patients clinical assessment. Paolo Livrea: Study desing and manuscript editing. Giulio E. Lancioni: Study design, manuscript editing.

### Conflict of interest statement

The authors declare that the research was conducted in the absence of any commercial or financial relationships that could be construed as a potential conflict of interest.
